# Analysis of sequence variations in low-density lipoprotein receptor gene among Malaysian patients with familial hypercholesterolemia

**DOI:** 10.1186/1471-2350-12-40

**Published:** 2011-03-19

**Authors:** Alyaa Al-Khateeb, Mohd K Zahri, Mohd S Mohamed, Teguh H Sasongko, Suhairi Ibrahim, Zurkurnai Yusof, Bin A Zilfalil

**Affiliations:** 1 Human Genome Centre, School of Medical Sciences, Universiti Sains Malaysia, Kubang Kerian, Kelantan, P.O. 16150, Malaysia; 2 Department of Medicine, School of Medical Sciences, Universiti Sains Malaysia, Kubang Kerian, Kelantan, P.O. 16150, Malaysia

## Abstract

**Background:**

Familial hypercholesterolemia is a genetic disorder mainly caused by defects in the low-density lipoprotein receptor gene. Few and limited analyses of familial hypercholesterolemia have been performed in Malaysia, and the underlying mutations therefore remain largely unknown.

We studied a group of 154 unrelated FH patients from a northern area of Malaysia (Kelantan). The promoter region and exons 2-15 of the LDLR gene were screened by denaturing high-performance liquid chromatography to detect short deletions and nucleotide substitutions, and by multiplex ligation-dependent probe amplification to detect large rearrangements.

**Results:**

A total of 29 gene sequence variants were reported in 117(76.0%) of the studied subjects. Eight different mutations (1 large rearrangement, 1 short deletion, 5 missense mutations, and 1 splice site mutation), and 21 variants. Eight gene sequence variants were reported for the first time and they were noticed in familial hypercholesterolemic patients, but not in controls (p.Asp100Asp, p.Asp139His, p.Arg471Gly, c.1705+117 T>G, c.1186+41T>A, 1705+112C>G, Dup exon 12 and p.Trp666ProfsX45). The incidence of the p.Arg471Gly variant was 11%. Patients with pathogenic mutations were younger, had significantly higher incidences of cardiovascular disease, xanthomas, and family history of hyperlipidemia, together with significantly higher total cholesterol and low density lipoprotein levels than patients with non-pathogenic variants.

**Conclusions:**

Twenty-nine gene sequence variants occurred among FH patients; those with predicted pathogenicity were associated with higher incidences of cardiovascular diseases, tendon xanthomas, and higher total and low density lipoprotein levels compared to the rest. These results provide preliminary information on the mutation spectrum of this gene among patients with FH in Malaysia.

## Background

Familial hypercholesterolemia (FH) is caused by defects in the low-density lipoprotein receptor (LDLR) gene, which give rise to a well-characterized clinical phenotype [1]. The prevalence of heterozygous FH is approximately 1 in 500 among the general population in most countries worldwide. The prevalence of homozygotes is generally about 1 in 1 million [1]. Despite its strong genetic background, FH shows great variability in phenotypic expression in terms of lipid profile, frequency of xanthomas, and onset and severity of cardiovascular disease (CVD) [1]. It has been shown that at least 50% of males and 20% of females with FH who fail to receive effective treatment suffer from coronary events by the age of 50 years [2].

The plasma levels of low-density lipoprotein cholesterol (LDL-C) in FH heterozygotes are lower and more dependent on other genetic and environmental factors than those in FH homozygotes. Although the nature of the molecular defect has some impact on the severity of hypercholesterolemia, FH heterozygotes with the same LDLR mutation can have widely different plasma levels of LDL-C. In all patients with FH caused by LDLR defects, phenotype severity seems to depend more on environmental factors than on the type of mutation [3]. However the clinical diagnosis is not always evident, especially in young patients without physical stigmata, and molecular diagnostic techniques would be helpful in these cases [4].

The LDLR gene locus is located on chromosome 19p13.1-13.3 and spans 45 kb, with 18 exons and 17 intervening introns, encoding a mature protein of 839 amino acids. Mutations can occur in the promoter, or in the introns or exons. The majority of variants fall within the ligand-binding (40%) or epidermal growth factor precursor-like (47%) domains, with the highest frequency of mutations reported in exon 4 (20%) [5]. This high frequency can be explained by a selection bias; i.e., individuals with functional mutations in this region may be overrepresented in the lipid clinic populations surveyed for FH screening [6]. However, when the number of mutations is normalized to base pairs, the mutation rate is similar in most exons, except for the promoter region and exons 16-18 [5].

A total of 1,066 individual validated LDLR variants from FH patients are now listed on the University College London (UCL) database [5]. The LDLR FH database http://www.ucl.ac.uk/ldl underlines the vast molecular heterogeneity of this disorder [5]. The current study therefore aimed to describe the molecular spectrum of FH in Malaysian subjects (by screening the most likely affected exons), and to identify and correlate the LDLR gene mutations with the clinical manifestations of FH.

## Results

One hundred and fifty-four patients were recruited. Their clinical characteristics and lipid profiles are presented in Table 1. Of these, 52.6% were females and 47.4% were males, with a mean age of 44.6 years. A high prevalence of cardiovascular disease (CVD) was reported (68.2%), with the mean age of presentation at 41.5 years. Around twenty-three percent of patients were current smokers. A family history of premature CVD (PCVD) was reported in 57.8%, and 58.4% had a family history of hyperlipidemia. Tendon xanthoma (TX) was present in 40.9%, and xanthelasma occurred in 25.3%. The mean levels of total cholesterol (TC) and LDL-C were 7.9 mmol/l and 5.0 mmol/l, respectively, while the TC: high-density lipoprotein (HDL) ratio without lipid-lowering therapy was 6.6. Eighty control subjects were recruited with a mean age of 39.3 years, a mean TC level of 5.0 mmol/l, and a mean LDL-C level of 2.9 mmol/l.

**Table 1:** Demographics, Clinical findings and Biochemical Data among FH patients.

Variables (n = 164)	
**A) Demographic characteristics**	

Age (year) *	44.6(12.0)

Sex n (%)	
Female	81(52.6)
Male	73(47.4)

Ethnicity n (%)	
Malays	152(98.7)
Non-Malays	2(1.3)

Smoking n (%)	
Current	36(23.4)
Ex-smokers	21(13.6)
Non-smokers	97(63.0)

Alcohol consumption n (%)	1(0.6)

**B)Medical history**	

CVD n (%)	105(68.2)
Age of onset of CVD(years)*	41.5(7.6)

Hypertension n (%)	34(22.1)

Stroke n (%)	4(2.6)

On LLT n (%)	126(81.2)

Family history of PCVD n (%)	89(57.8)
Family history of hyperlipidemia n (%)	90(58.4)
Family history of tendon xanthoma n(%)	48(31.2)

**C) Medical examination**	

SBP (mmHg) *	127.1(15.9)
DBP (mmHg)*	77.7(10.9)

BMI (kg/m2)*	27.4(4.8)

Tendon xanthomas n(%)	63(40.9)

Arcus n (%)	77(50.0)

Xanthelasma n (%)	39(25.3)

**D) Lipid profile parameters**	

TC(mmol/l)*	7.9(1.0)
TG (mmol/l)*	1.9(0.8)
HDL-C (mmol/l)*	1.3(0.3)
LDL-C (mmol/l)*	5.0 (0.9)
TC/HDL-C ratio *	6.6(2.3)

*The data are expressed as mean (S.D).

LDLR gene variants were identified by analyzing the promoter region of the LDLR gene and the exon-intron boundaries of exons 2-15 using denaturing high-performance liquid chromatography (DHPLC), and heteroduplex peaks were sequenced. Table 2 shows the different sequence variants that were identified in 154 unrelated FH patients attending the Cardiology Clinic in the Hospital Universiti Sains Malaysia (HUSM), Kelantan, Malaysia.

**Table 2:** LDLR gene variants with in-Silico analysis on the effect of the variants.

Variant name*	Location	In Silico analysis		Statistical analysis			Miscellaneous	Reference
		
		Non-pathogenic	Pathogenic	Patient n(%)^a^	Control n(%)^b^	** P value		
Nil	promoter							

c.81C>T;p.Cys27Cys	exon 2	√		1(0.6)			rs2228671	NCBI; UCL***

c.190+56G>A	Intron 2	√		3(1.9)			rs3745677	NCBI; UCL***

c.190+58C>T	intron 2	√		7 (4.5)	4(5)	0.9	rs3745678	NCBI

c.300C>T;p.Asp100Asp	exon 3	√		1(0.6)			Synonymous Unlikely to cause pathogenicity	**No reference**

c.910G>A;p.Asp304Asn	exon 6	√		7(4.5)			Changes in amino acid Benign by Poly Phen	UCL***

c.940+36G>A	intron 6	√		3(1.8)			rs13306513	NCBI

c.1060+7T>C	intron 7	√		18(11.7)	8(10)	0.7	rs2738442	NCBI

c.1060+10G>C	intron 7	√		5(3.2)	3(3.8)	0.9	rs12710260	NCBI; HGMD****

c.1186+41T>A	intron 8	√		1(0.6)			Location is unlikely to cause pathogenicity	**No reference**

c.1194C>T;p.Ile398Ile	exon 9	√		5(3.2)			rs13306498	NCBI

c.1359-30C>T	intron 9	√		2(1.3)			rs1003723	NCBI

c.1411A>G;p.Arg471Gly	exon 10	√		17(11.0)			Changes in amino acid Benign by Poly Phen	**No reference**

c.1617C>T;p.Pro539Pro	exon 11	√		6 (3.9)	4(5)	0.7	rs5929	NCBI

c.1705+56C>T	intron 11	√		12 (7.8)	2 (2.5)	0.08	rs4508523	NCBI

c.1705+112C>G	intron 11	√		1(0.6)			Location is unlikely to cause pathogenicity	**No reference**

c.1706-55A>C	intron 11	√		6 (3.9)			rs2738447	NCBI

c.1706-69G>T	intron 11	√		1(0.6)			rs7259278	NCBI

c.1705+117T>G	intron 11	√		2(1.3)			Location is unlikely to cause pathogenicity	**No reference**

c.1773C>T;p.Asn591Asn	exon 12	√		7(4.5)			rs688	NCBI

c.1959T>C;p.Val653Val	exon 13	√		1(0.6)			rs5925	NCBI

c.2232A>G;p.Arg744Arg	exon 15	√		5 (3.2)	3(3.8)	0.8	rs5927	NCBI

c.190+4A>T	intron 2		√	1(0.6)			Possibly pathogenic	UCL**

c.301G>A;p.Glu101Lys	exon 3		√	11(7.1)			Possibly pathogenic	UniProt; UCL***

c.415G>C;p.Asp139His	exon 4		√	1(0.6)			Probably pathogenic	**No reference**

c.601G>A;p.Glu201Lys	exon 4		√	9(5.8)			Probably pathogenic	UCL**

c.763T>A;p.Cys255Ser	exon 5		√	10(6.5)			Probably pathogenic	UCL***

c.1706_1845dup;p.Asp616IlefsX96	exon 12		√	2(1.3)			Pathogenic	**No reference**

c.2100C>G;p.Asp700Glu	exon 14		√	5(3.2)			Possibly pathogenic	UniProt; UCL***

c.1996_2012del17;p.Trp666ProfsX45	exon 14		√	4(2.6)			Pathogenic	**No reference**

*Naming of the variants done according to Nomenclature of the Human Genome Variation Society (HGVS).

** Chi square test

*** http://www.ucl.ac.uk/Library/database

****Human Genome Mutation Database

*****Nonsense-mediated decay

rs reference sequence from NCBI

^a ^percentage from 154

^b ^percentage from 80**

Screening of all clinically diagnosed cases with FH identified 29 LDLR variants in 117 of 154 subjects included in the study (76.0%). Six of the variants were detected in the control group, with a non-significant difference in frequency between FH subjects and controls (c.190+58C>T, c.1060+7 T>C, c.1060+10 G>C, p.Pro539Pro, c.1705+56C>T and p.Arg744Arg). All of the variants were reported to occur in a heterozygous state, except for c.2232A>G; (p.Arg744Arg) in exon 15 and c.1060+7 T>C in intron 7, which were homozygous.

The most frequent variant was the c.1060+7 T>C, with a frequency of 11.7% (18/154), followed by the c.1411A>G substitution in exon 10, resulting in p.Arg471Gly variant, which was detected at a frequency of 11.0% (17/154).

Eight variants were previously unreported while the other 21 variants have been previously described in public databases http://www.hgmd.cf.ac.uk; http://www.ucl.ac.uk/ldlr, and http://www.ncbi.nlm.nih.gov/Genbank (Table 2).

There was considerable diversity in the types of variants, as eight LDLR gene mutations were reported (1 frameshift, 1 large rearrangement, 5 missense and 1 splice site).

**Figure 1:**
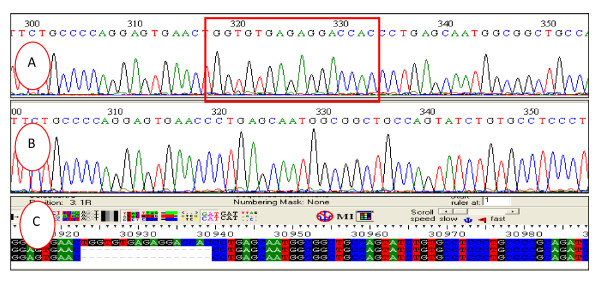
**Sequencing analysis demonstrated the detection of Trp666ProfsX45 mutation in exon14**. A) Nucleotide sequences are shown by chromatogram before deletion (red square shows the deleted region, B) Nucleotide sequences are shown by chromatogram after deletion C) Nucleotide sequences are shown by alignment with normal exon 14 deletion at 30923-30939.

One novel frameshift mutation was reported, p.Trp666ProfsX45 (Figure 1), resulting from the deletion of 17 nucleotides (c.1996_2012del 17). A novel silent variant was reported in one FH subject (p.Asp100Asp). Two novel missense variants were also reported, p.Asp139His and p.Arg471Gly.

**Figure 2:**
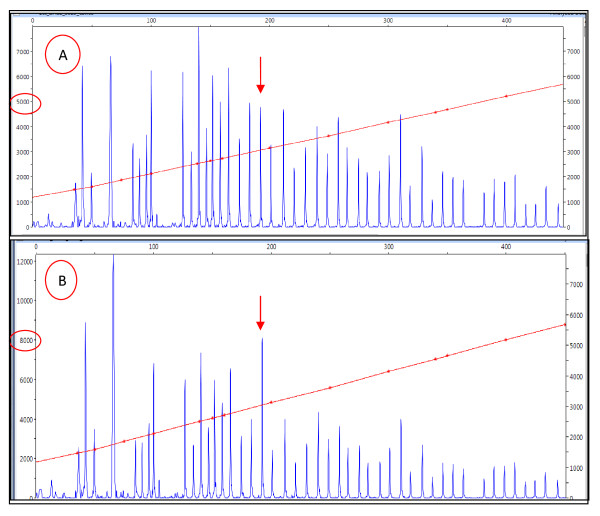
**A- The multiplex ligation-dependent probe amplification kit of control subject**. The red arrow shows peak height of exon 12 probe of 4768 RFU. B-A case of a duplication of exon 12 probe. The red arrow shows the peak height of 8084 RFU. The data were analyzed using the Peak Scanner software.

A duplication of exon 12 was found to co-segregate with hypercholesterolemia in two family members. A typical result of duplication is shown in the electrophoregram in Figure 2A and 2B, which shows the peak height for each of the test and control probes. The 37 labeled peaks included probes for the LDLR gene promoter, its 18 exons, 16 reference probes, and 2 probes for the upstream and downstream genes in chromosome 19. The peak height that represents the exon 12 probe was at 8,084 relative fluorescence units (RFU), which was about 1.7 times that of the control sample (4,768 RFU), suggesting duplication. This result was confirmed by second multiplex ligation-dependent probe amplification (MLPA).

The c.190+4A>T splice site mutation was reported in one FH subject.

Three previously reported silent variants, p.Ile398 Ile, p.Asn591Asn, and p.Val653Val, were found in the FH subjects but not in the control group, while one silent variant (p.Pro539Pro) was reported in both cases and controls. One novel variant in intron 8 (c.1186+41T>A) and two previously unreported variants in intron 11 (c.1705+117T>G and c.1705+112C>G) were also noticed.

### In silico analyses of variant effects

We categorized 21 variants as non-pathogenic (Table 2). Alamut™identified fifteen of them as "might be polymorphism" with their designated SNP ID from the NCBI. Another software, Polymorphism phenotyping (PolyPhen), predicted two other exonic variants (c.910G>A;p.Asp304Asn and c.1411A>G;p.Arg471Gly) to be "benign", and these were therefore also regarded as non-pathogenic. We also categorized a synonymous variant, c.300C>T;p.Asp100Asp, as non-pathogenic because no evidence of splicing aberrations or changes in protein structure from the in-silico analyses although it may stall translation by requiring the use of low abundance tRNAs. We were unable to predict the pathogeniciy of three intronic variants (c.1186+41T>A, c.1705+117T>G, c.1705+112C>G). However, due to their positions, they seem unlikely to cause pathogenicity and therefore were categorized as non-pathogenic.

Eight variants were categorized as pathogenic, in which the detailed in-silico analyses are provided in Table 3. Of these, two (c.1706_1845dup;p.Asp616IlefsX96 and c.1996_2012del;p.Trp666ProfsX45) were categorized as pathogenic based on their frameshift effects. Two others (c.301G>A; p.Glu101Lys and c.2100C>G;p.Asp700Glu) were categorized as ''possibly pathogenic'' based on Uni-Prot reports. Another one (c.190+4A>T) was categorized as possibly pathogenic based on further analyses using Analyzer Splice Tool (AST) and NNSPLICE softwares. According to Saphiro and Senapathy (1987) [7], it occurred within a conserved 5'splice-donor site. However, its true effects have yet to be clarified with an mRNA analysis. Three exonic mutations (c.601G>A; p.Glu201Lys, c.763T>A;p.Cys255Ser and c.415G>C;p.Asp139His) were predicted by Poly Phen software as "probably damaging" and were therefore regarded as pathogenic mutations (Table 3).

**Table 3:** The predicted pathogenic variants

Name of Variant	Location	Analysis by NNSPLICE	Analysis by AST	Alamut	Uni prot report	Poly Phen
c.190+4A>T	Intron 2	Before mutation the score was 0.54. After mutation,not recognized as splice site	Before change the score was 70.3 and ΔG -3.6 After mutation the score was 60.32 and ΔG -3.0			

c.301G>A;p.Glu101Lys	Exon 3			Possibly pathogenic as reported by UniProt (FTId: VAR_005315)	Change from medium size and acidic (E) to large size and basic (K)	

c.415G>C;p.Asp139His	Exon 4					Changes in amino acid. Disruption of ligand binding site

c.601G>A;p.Glu201Lys	Exon 4					Changes in amino acid. Disruption of ligand binding site

c.763T>A;p.Cys255Ser	Exon 5					Changes in amino acid. Disruption of ligand binding site

c.1706_1845dup;p.Asp616IlefsX96	Exon 12			Pathogenic	This duplication creates a frame shift starting at codon Asp616. The new reading frame ends in a STOP codon 95 positions downstream. The mRNA produced might be targeted for nonsense mediated decay (NMD).	

c.1996_2012del;p.Trp666ProfsX45	Exon 14			Pathogenic	This deletion creates a frame shift starting at codon Trp666. The new reading frame ends in a STOP codon 44 positions downstream. The mRNA produced might be targeted for nonsense mediated decay (NMD).	

c.2100C>G;p.Asp700Glu	Exon 14			Possibly pathogenic as reported by UniProt (FTId: VAR_005412)	Change in 3 D structure of protein	

Six previously unreported variants were submitted to GenBank and assigned accession numbers (Table 4).

**Table 4:** Novel variants of LDLR gene that were submitted to genbank and their accession numbers.

Variant name	Accession no.
p.Asp100Asp	HM853677

p.Asp139His	HM853677

p.Arg471Gly	HQ190922

c.1705+117 T>G	HQ190924

c.1186+41T>A	HQ190917

p.Trp666ProfsX45	HM369522

### Phenotypes of FH subjects with identified LDLR gene variants

In order to evaluate the relevance of LDLR pathogenic mutations to the clinical presentation of the disease, individuals were classified into two groups according to the pathogenicity of the gene defect: those with non-pathogenic variants (n = 112) were compared to those with pathogenic mutations (n = 42) (Table 5). Patients with pathogenic mutations were younger (P = 0.007), have a higher frequency of CVD (P = 0.001) with younger age of presentation (P = 0.03), higher frequency of family history of hyperlipidemia (P = 0.03), xanthomas (P < 0.001), and higher levels of TC (P = 0.001) and LDL-C (P = 0.02).

**Table 5:** Association of LDLR sequence variants among patients to their demographic characteristics, medical history, clinical finding and lipid profile parameters (n = 154)

Variables (n = 164)	Patients with non- pathogenic variants (n = 112) (72.7%)	Patients with pathogenic variants (n = 42) (27.3%)	P value
**A)Demographic characteristics**			
Age (year)*^a^	46.2(11.4)	40.3(12.7)	0.007
Sex	60(53.6)	21(50.0)	0.72
Female	52(46.4)	21(50.0)	
Male			

**B) Medical history**			
CVD (%)^b^	68(60.7)	37(88.1)	0.001
Age of onset of CVD(%)^b ^**	42.7(6.4)	39.3(9.3)	0.03
Hypertension (%)^b^	30(26.8)	4(9.5)	0.03
Family history of PCVD (%)^b^	60(53.6)	29(69.0)	0.1
Family history of hyperlipidemia (%)^b^	59(52.7)	31(73.8)	0.03
Family history of tendon xanthoma (%)^b^	32(28.6)	16(38.1)	0.3

**C) Clinical examination**			
BMI	27.1(4.5)	28.2(5.5)	0.25
Tendon xanthoma	35(31.3)	28(66.7)	<0.001
(%)^b^Arcus	57(50.9)	20(47.6)	0.9
(%)^b^Xanthelasma (%)^b^	24(21.4)	15(35.7)	0.09

**D) Lipid profile parameters^a^**			
TC(mmol/l)*	7.7(0.9)	8.3 (1.2)	0.001
TG (mmol/l)*	1.9(0.8)	1.7(0.9)	0.19
HDL-C (mmol/l)*	1.3(0.3)	1.3(0.4)	0.96
LDL-C (mmol/l)*	4.9(0.8)	5.2(1.1)	0.02
TC/HDL-C ratio *	6.5(2.4)	6.9(1.9)	0.23

*Data are expressed as mean (S.D).

^a ^ANOVA test was used

^b ^Chi square test was used

^c ^The pathogenic mutations are reported 43 times according to Table 2 and these were found among 42 patients as one patient was reported to have 2 pathogenic mutations [Glu101Lys+Dup exon 12].

## Discussion

Molecular genetic diagnosis is currently the most specific method for identifying patients with FH. Although numerous LDLR gene mutations have been identified in FH patients, genetic data for the Malaysian population are rare [4,8]. The present study cohort of 154 patients with clinical features of FH was relatively young (with a mean age of 44.6 years), and already had a high prevalence of CVD (68.2%). The average LDL-C level was classified as high, according to National Cholesterol Education Program (NCEP ATP III) classification [9]. This, together with the high prevalence of CVD, were consistent with the recognized association of elevated LDL-C levels with a high risk of atherosclerotic disease [10].

The presence of gene sequence variants in the promoter region and in exons 2-15 of the LDLR gene was investigated, and LDLR variants were identified in 117 out of 154 patients (76.0%). These comprised 8 previously unreported and 21 previously reported LDLR sequence variations. Eight pathogenic mutations were reported, including one intronic (c.190+4A>T).

One subset was relatively common, such as a variant in exon 10 (p.Arg471Gly), which was found in 17 patients. The donor splice site mutation (c.190+4A>T) was the commonest among Filipino FH [11], while L547V mutation was predominant among Japanese FH [12]. The frequent detection of a deleterious mutation may be the result of consanguinity, recurrent mutational events, genetic drift, or multiple introduction of the mutation into a population [13].

The overall mutation rate of 42.2% (65/154), which was higher than that reported in previous studies of Malaysian FH (26%) [4] or Filipino FH (20%) patients [11], but lower than that reported in European populations (52%) [14].

Only the p.Cys255Ser, p.Asn591Asn, and p.Val653Val variants detected in the current study have previously been reported among Malaysian FH subjects [8]; the remainders were newly identified in this study population. This may be attributed to the high sensitivity of DHPLC for detecting gene sequence variants, and the restricted inclusion criteria.

Five missense mutations that resulted in altered amino acids (p.GluA101Lys, p.Asp139His, p.Glu201Lys, p.Cys255Ser and p.Asp700Glu) were predicted to exert pathogenic effects based on the in silico analyses.

p.Glu101Lys was predicted to be pathogenic by Alamut™software with change from medium size and acidic (Glutamate) to large size and basic (Lysine). This residue occurs in the second disulfide-rich repeat in the binding domain of the receptor protein, and affects processing and intracellular transport of the newly synthesized protein. It was suggested that the normal formation of disulfide bonds in the second repeat may be impeded in the mutant protein [15].

p.Asp700Glu was predicted to be pathogenic by Alamut™software with change in the 3 D structure of the protein. It was reported among Spanish FH subjects [16].

p.Asp139His is a missense mutation, reported for the first time in this study, located in the ligand binding domain and was predicted to be ''probably damaging'' by PolyPhen due defect in the ligand binding site.

p.Glu201Lys and p.Cys255Ser are previously reported mutations among Russian patients [17] and Malaysian patients [8], respectively and were predicted to be ''probably damaging'' by PolyPhen.

It has been reported that a significant proportion (approximately 18.9%) of the gene sequence variants observed in patients with FH have no effect on the protein coding sequence [18]. These variations are often located in the 5' splice-donor site or the 3' splice-acceptor site of an intron, and are predicted to result in either exon skipping or retention of an intron in the mRNA, although this has not always been confirmed experimentally. One such variant, c.190+4A>T, has been reported in this study as well as in the Netherlands [19] and the Philippines [11]. We reported the possibility that it may cause splicing abnormalities through an in-silico analysis. However, the true effect should be experimentally clarified using splicing analysis.

For c.1060+10G>C variant in intron 7, it was reported in French and Greek FH patients [20,21]. In the present study this variant was reported among FH subjects and control groups with a non significant difference in the occurrence between both groups which may indicate that this variant may be a polymorphism.

c.1359-30C>T is detected in unrelated FH individuals in Denmark [22]. Webb et al. [23] failed to detect any effect of this polymorphism on plasma lipid concentrations in their population. p.Ile398 Ile, p.Pro539Pro, and p.Cys27Cys are previously reported among European [24], Russian [25] and Chinese FH patients [26], respectively, with unknown clinical associations with the disease.

p.Trp666ProfsX45 was identified in four unrelated patients within this study, which could be attributed to a founder effect. The extent or nature of the deletion in the DNA was not clear, and the amplified mutant fragment was therefore cloned and sequenced. The predicted translation products of the alleles carrying this mutation resulted in the creation of premature stop codon downstream of the deletion.

We identified a large genomic rearrangement (c.1706_1845dup;p.Asp616IlefsX96), a duplication of exon 12, in the LDLR gene which have not previously been detected among Malaysian FH patients [8,27]. It is interesting to note that while large deletion generally represents 85% of large rearrangements in the LDLR gene [5], our finding showed the predominance of a large duplication and the absence of large deletions. Large duplications seem to be much more prevalent than large deletions among the Malaysian population, while large deletions are more common than large duplications in North European Caucasians [28]. Therefore, the prevalence of large duplications/rearrangements in FH patients indicates that MLPA should be included in the diagnostic service for dominant hypercholesterolemia.

The duplicated exon 12 (c. c.1706_1845dup; p.Asp616IlefsX96) is predicted to cause a frameshift protein, with premature termination 95 positions downstream. Due to its nucleotide number (1706-1845 bp) that were duplicated, either deletion or duplication of exon 12 will cause frameshifted protein, (i.e. disruption of reading frame starting from the point of deletion or duplication). The mutation effect is different with that of previously reported deletion of exon 15 [29] which is a common yet mild cause of FH in Finland. Deletion of exon 15 caused only internal truncation of the protein without inducing premature termination, since the reading frame was maintained.

In silico predictions of the eight variants categorized as pathogenic may provide grounds for further experimental studies aimed at revealing mRNA abnormalities, as well as altered protein-protein interactions and loss of function.

mRNA expression studies could be performed for the c.1706_1845dup; p.Asp616IlefsX96 and c.1996_2012del;p.Trp666ProfsX45, as they were also predicted to be targets for nonsense-mediated decay.

There were significant differences in baseline LDL-C levels between the two patients groups; subjects with pathogenic mutations had a mean baseline LDL-C level of 5.2 mmol/l, compared with level of 4.9 mmol/l in patients of the non pathogenic group.

Also those with pathogenic mutations show a more severe phenotype (higher frequencies of CVD, TX, and family history of hyperlipidemia) than those with non pathogenic variants which may increase the likelihood of their pathogenicity. The exact decision about the functional implications should be done by in vitro functional study.

The presentation of TX as a diagnostic sign of FH was interesting in the current study population, as only 40.9% of FH subjects showed TX. This emphasizes the importance of DNA analysis, because no definite diagnosis of FH can be made without the identification of TX or a defined mutation.

Regarding those patients who showed definite clinical manifestations of FH but no identified LDLR sequence variations, the following possibilities exist: (1) they may have defects that are linked to the LDLR gene variant within deep intronic regions, affecting expression or splicing of the gene; (2) they may have exon defects that have not been screened; (3) the defect may lie in another gene involved in LDL-C metabolism, such as the apolipoprotein gene or the proprotein convertase subtilisin/kexin type 9 (PCSK 9) gene; or (4) it is possible that polygenic factors interacting with environmental factors may lead to a clinical diagnosis mimicking the FH phenotype [30]. It is also likely that some patients with no identified mutation do not have true monogenic autosomal dominant hypercholesterolemia. The clinical diagnostic criteria for FH are not precise, and no family studies have been carried out in patients with any detected mutations to clarify this.

There were several limitations of the present study. FH patients are generally seen by cardiologists when cardiovascular complications appear, but the disease's etiology is not always investigated. This could explain the low number of FH patients recruited in the study sample. This cohort was therefore representative of FH patients who are referred to specialists because of a severe lipid phenotype, but not of those who remain undiagnosed, who may have milder phenotypes [31]. The DHPLC method used here has been reported to show a sensitivity of 96% [32], and an additional 4% of LDLR mutations may thus have remained undetected. Furthermore, several exons with a reportedly low mutation detection rate were not examined; the detection rate may have been increased by a further 3-4% (representing the percentage of mutations in exon 1, 16,17 and 18 [5] if all exons had been included. Additionally, only 30-50 base pairs of the LDLR introns were examined by the primers used here, and mutations may exist in other regions, although few have been reported [5].

## Conclusion

In summary, 29 LDLR gene sequence variants were detected in 76% of the analyzed patients with a clinical diagnosis of FH. Most of these variants were reported for the first time among Malaysians, with eight variants discovered in this report. Patients with pathogenic mutations had a higher rate of CVD and higher TC and LDL-C levels than those with non-pathogenic variants. These findings support the usefulness of genetic testing in FH, beyond its ability to provide an unequivocal diagnosis of the condition.

## Methods

The target population included dyslipidemic patients attending the Cardiology Clinic in HUSM. One hundred and fifty-four subjects gave written, informed consent to participate in this study. The included subjects were patients who fulfilled the Simon Broome Familial Hypercholesterolemia Register diagnostic criteria for FH [2]. Patients with suspected causes of secondary hyperlipidemia were excluded from the study [33]. Demographic data, medical history, lipid-lowering therapy, physical examination for detection of the presence of TXs, xanthelasma, and arcus cornealis were recorded. Pre-treatment lipid profiles were obtained from the patients' records and family history of PCVD was obtained from all patients using a standardized form.

Eighty control subjects were recruited to detect the presence of nucleotide substitutions that could be regarded as single nucleotide polymorphisms. The control subjects were randomly chosen healthy volunteers from the staff of Universiti Sains Malaysia, who had no previous history or family history of hyperlipidemia or PCVD, no history of secondary causes of hyperlipidemia and no any clinical signs of hyperlipidemia. Ethical approval for the study was obtained from Universiti Sains Malaysia's Research and Ethics Committee.

### Molecular analysis

Peripheral venous blood (2 ml) was collected from patients and controls into tubes containing potassium ethylenediaminetetraacetic acid, and genomic DNA was extracted by (Qiagen, Hilden, Germany) kit. The samples were then stored at -20°C until further analysis. A further 3 ml was collected and analyzed to exclude secondary causes of hyperlipidemia.

All patient and control subjects were screened for mutations within the LDLR gene. Thirteen pairs of primers based on the LDLR reference sequence obtained from the GenBank database (accession no. NT_011295) were designed to cover the regions previously shown to contain the highest prevalence of mutations [5] (promoter region and exons 2-15), including 30-50 bp regions of the introns covering the exon-intron junctions to detect potential splice site mutations. Briefly, samples were analyzed by polymerase chain reaction (PCR) standardized using genomic DNA and primer pairs to amplify the target exons. PCR was performed in 20 μl reactions. Each reaction contained 0.5 μM of each forward and reverse primer, 0.16 mM dNTPs, 2.5 mM MgCl_2_, 1 × PCR buffer, 0.75 U Taq DNA polymerase and diluted DNA template (~50 ng). Details of the primer sequences, annealing temperatures, and expected PCR products are available in Additional file 1. Before DHPLC analysis, 5 μl of the PCR reaction mixture was run on a 2% agarose gel with a 100-bp marker for comparison.

### DHPLC and direct sequencing

All patients and controls were screened for point mutations using DHPLC. Mutation analysis was performed using a partially inert Helix System (Varian, Inc, USA). The melting temperatures of the DNA fragments were predicted using the procedure discussed at http://insertion.stanford.edu./melt.html. The PCR products were denatured at 95°C for 5 min and cooled to 65°C at a rate of 1°C/min. After slow re-annealing, the sample was injected directly at the optimum temperature identified by mapping, and the elution profile of the samples was compared to the elution profile of the control. The single peak pattern of the sample under partial denaturation conditions indicated the absence of mismatch, and thus identified mutations. In contrast, samples that demonstrated different peak patterns were considered to be heteroduplex gene variants, and bidirectional sequencing of purified PCR products from these cases was performed to confirm the results of DHPLC. PCR fragments were sequenced using the ABI PRISM BigDye terminator cycle model ABI Prism 3100 Genetic Analyzer (Applied Biosystems, California, USA), according to the manufacturer's recommendations.

### Multiplex Ligation Dependent Probe Amplification (MLPA)

To detect large rearrangements within the coding sequence of the LDLR gene, samples were analyzed using MLPA, in accordance with the manufacturer's protocol (Kit P062, MRC-Holland, Amsterdam, The Netherlands). At least five control samples were analyzed simultaneously. The PCR products were fractionated on an ABI sequencer. The data were retrieved using Peak Scanner software (v1.0), and exported to a Microsoft Excel spreadsheet for further analysis of peak heights. The peak heights of non-ligated probes were negligible compared with those of the probe fragments. The presence of a sequence variant was confirmed by a second MLPA analysis. The expected normalized values were 1.0, 0.5, and 1.5 for normal, heterozygous deletion, or duplication, respectively [34].

### In silico analyses of variant effects

Online computer programs were used to analyze the effects of the nucleotide substitutions. All the variants were subjected to in silico analyses using Alamut™v1.54 (Interactive Biosoftware, Rouen, France) [35], which screened for splicing abnormalities as well as protein changes. Variants without identifiable pathogenicity were further categorized into those located within splice sites [7] and those located within exons and beyond splice sites. Variants located within splice sites were further analyzed using the online software NNSPLICE v0.9 [36]http://www.fruitfly.org/seq_tools/splice.html, with a cutoff value of 0.4, and AST [7,37,38]http://ibis.tau.ac.il/ssat/Splice SiteFrame.htm, to predict changes in the splice sites. Variants located within exons in which Alamut™could not predict the pathogenicity were subjected to analysis with Poly Phen [39], which is an automatic tool for predicting the possible impact of an amino acid substitution on the structure and function of a human protein. It classifies amino acid substitutions as probably damaging, possibly damaging, or benign, and provides position-specific independent count scores for wild-type and variant proteins mapped to a known 3 D structure http://genetics.bwh.harvard.edu/pph/[39].

Nucleotide numbers were designated using the LDLR sequence reported at http://www.ucl.ac.uk/fh, with cDNA numbering beginning with A of ATG = 1. The mutations were designated following the system of the Human Genome Variation Society http://www.hgvf.org.

Mutation was defined as sequence change which is clearly designated as FH causing, such as frameshifts and those predicted to be pathogenic by computer programmes. The rest of the variants were simply called as variants.

### Statistical analysis

The distributions of quantitative variables were tested for normality. An initial descriptive analysis was carried out using number of cases and percentages for qualitative variables and mean (SD) for quantitative variables with a normal distribution. Quantitative variables were compared using independent t-test, and qualitative variables were analyzed using chi-squared tests. Genotype frequencies were also compared using chi-squared tests. A global significance level of P < 0.05 was used for all analyses. All statistical analyses were performed with SPSS software (v13.0, SPSS, Inc., Chicago, Illinois). Pre-treatment levels of TC, HDL-C, triglycerides, and LDL-C and TC/HDL-C ratio were used for statistical analysis.

## Competing interests

The authors declare that they have no competing interests.

## Authors' contributions

ALKH collected the samples, carried out PCR, DHPLC, sequencing, MLPA, analyzed clinical samples, data analysis and drafted the manuscript. MKZI designed the primers, contributed to the PCR optimization. MSM was involved in the initial study design, in protocol development and selection of patients. THS contributed to the in-silico analyses of the mutation effects and manuscript revision. SI was involved in the initial study design, in protocol development and selection of patients. ZY was involved in the initial study design, in protocol development. BAZ was involved in the initial study design, in protocol development, the manuscript revision and is the team leader. All authors read and approved the final manuscript.

## Pre-publication history

The pre-publication history for this paper can be accessed here:

http://www.biomedcentral.com/1471-2350/12/40/prepub

## Supplementary Material

Additional file 1 **Supplementary on line materials**. Click here for file
