# Supplementary material for: Analysis of sequence variations in low-density lipoprotein receptor gene among Malaysian patients with familial hypercholesterolemia
Source: BMC Med Genet. 2011 Mar 19;12:40. doi: 10.1186/1471-2350-12-40 (PMC3071311; doi:10.1186/1471-2350-12-40)
Supplement: Additional file 1 — Supplementary on line materials. [file 1471-2350-12-40-S1.DOCX]

**Additional files**

Additional file 1

Title: Primer sequence, melting, annealing temperatures and product size.

Description: the following table contains the sequence of each primer used, melting, annealing temperatures and the product size of each PCR product.

| **Primer name** | **Primer sequence** | **Tm** | **Ta** | **Size(bp)** |
| --- | --- | --- | --- | --- |
| Promoter region F | 5´CAGCTCTTCACCGGAGACC´3 | 62 | 65 | 277 |
| Promoter region R | 5´ACCTGCTGTGTCCTAGCTGG´3 | 64 |  |  |
| Exon 2 F | 5´CTGATTCTGGCGTTGAGAG´3 | 58 | 63 | 279 |
| Exon 2 R | 5´GAGGCCAGCGGATCACTT ´3 | 58 |  |  |
| Exon 3 F | 5´GAGTGACAGTTCAATCCTG ´3 | 56 | 64 | 298 |
| Exon 3 R | 5´GAAGAGGCTTGGTATGAGC´3 | 58 |  |  |
| Exon 4 F | 5´GAGACTTCACACGGTGATG ´3 | 58 | 61 | 478 |
| Exon 4 R | 5´CCCAGGGACAGGTGATAG´3 | 58 |  |  |
| Exon 5F | 5´ TCTGGTTGTCTCTTCTTGAG´3 | 58 | 62 | 229 |
| Exon 5 R | 5´ TGCAAGCAGCAAGGCACA ´3 | 56 |  |  |
| Exon 6 F | 5´ TCAGACACACCTGACCTTC´3 | 58 | 60 | 312 |
| Exon 6 R | 5´ CCGTGCGAGACTGTCTCA ´3 | 58 |  |  |
| Exon 7 F | 5´GTTGTAATGAGCCAAGGTTG´3 | 58 | 63 | 316 |
| Exon 7 R | 5´CTCCTAACTGCTTTCAAGCA´3 | 58 |  |  |
| Exon 8 F | 5´TCTCCTGGCTGCCTTCGAA ´3 | 60 | 63 | 429 |
| Exon 8 R | 5´CTAGGACATATGCAGGCATC ´3 | 60 |  |  |
| Exon 9 F | 5´CACTCTTGGTTCCATCGAC ´3 | 58 | 63 | 620 |
| Exon 10 R | 5´CCACTAACCAGTTCCTGAA ´3 | 56 |  |  |
| Exon 11F | 5´CTTCCAGAATTCGTTGCAC´3 | 56 | 63 | 358 |
| Exon 11 R | 5´ACAGACCAAGACCTCATCT ´3 | 56 |  |  |
| Exon 12 F | 5´GTTCAGGCTCACATGTGGTT´3 | 60 | 63 | 374 |
| Exon 12 R | 5´GTTCATCTTGGCTTGAGTG ´ 3 | 56 |  |  |
| Exon 13 F | 5´GTGGCCTGTGTCTCATCCCA ´3 | 64 | 63 | 560 |
| Exon 14 R | 5´GAGCAGAGAGAGGCTCAGGA´3 | 60 |  |  |
| Exon 15 F | 5´TAGGCGCACACCTATGAGAA´ 3 | 60 | 60 | 348 |
| Exon 15 R | 5´GTGAGAGAAGGTCAGCAAGG´3 | 58 |  |  |

F: forward primer, R: Reverse primer, Tm: Melting temperature, Ta: Annealing temperature
